# 2'-Deoxythymidine Adducts from the Anti-HIV Drug Nevirapine

**DOI:** 10.3390/molecules18054955

**Published:** 2013-04-26

**Authors:** Alexandra M. M. Antunes, Benjamin Wolf, M. Conceição Oliveira, Frederick A. Beland, M. Matilde Marques

**Affiliations:** 1 Centro de Química Estrutural, Instituto Superior Técnico, Universidade Técnica de Lisboa, Av. Rovisco Pais, 1049-001 Lisboa, Portugal; 2 Division of Biochemical Toxicology, National Center for Toxicological Research, Jefferson, AR 72079, USA

**Keywords:** nevirapine, non-nucleoside reverse transcriptase inhibitor, carcinogenicity, DNA adducts, palladium catalysis

## Abstract

Nevirapine (NVP) is a non-nucleoside reverse transcriptase inhibitor (NNRTI) used against HIV-1. Currently, NVP is the most widely used anti-HIV drug in developing countries, both in combination therapy and to prevent mother-to-child transmission of HIV. Despite its efficacy against HIV, NVP produces a variety of toxic responses, including hepatotoxicity and skin rash. It is also associated with increased incidences of hepatoneoplasias in rodents. In addition, epidemiological data suggest that NNRTI use is a risk factor for non-AIDS-defining cancers in HIV-positive patients. Current evidence supports the involvement of metabolic activation to reactive electrophiles in NVP toxicity. NVP metabolism includes oxidation to 12-hydroxy-NVP; subsequent Phase II sulfonation produces an electrophilic metabolite, 12-sulfoxy-NVP, capable of reacting with DNA to yield covalent adducts. Since 2’-deoxythymidine (dT) adducts from several alkylating agents are regarded as having significant mutagenic/carcinogenic potential, we investigated the formation of NVP-dT adducts under biomimetic conditions. Toward this goal, we initially prepared and characterized synthetic NVP-dT adduct standards using a palladium-mediated Buchwald-Hartwig coupling strategy. The synthetic standards enabled the identification, by LC-ESI-MS, of 12-(2'-deoxythymidin-N3-yl)-nevirapine (N3-NVP-dT) in the enzymatic hydrolysate of salmon testis DNA reacted with 12-mesyloxy-NVP, a synthetic surrogate for 12-sulfoxy-NVP. N3-NVP-dT, a potentially cytotoxic and mutagenic DNA lesion, was also the only dT-specific adduct detected upon reaction of dT with 12-mesyloxy-NVP. Our data suggest that N3-NVP-dT may be formed *in vivo* and play a role in the hepatotoxicity and/or putative hepatocarcinogenicity of NVP.

## 1. Introduction

The use of non-nucleoside reverse transcriptase inhibitors (NNRTIs) as components of initial combined antiretroviral therapy (CART) is recommended by the World Health Organization guidelines on HIV/AIDS [1]. However, epidemiological studies on the incidence of non-AIDS defining cancers in individuals undergoing CART have suggested that the use of NNRTIs is a risk factor [2]. This raises concerns about the chronic administration of this class of drugs, particularly in pediatric settings. Moreover, taking into account the substantially higher life expectancy and quality of life that HIV-infected patients currently achieve, understanding the molecular basis of NNRTI-induced toxicities is essential to develop accurate risk/benefit estimations that can guide decisions on treatment options.

**Figure 1:**
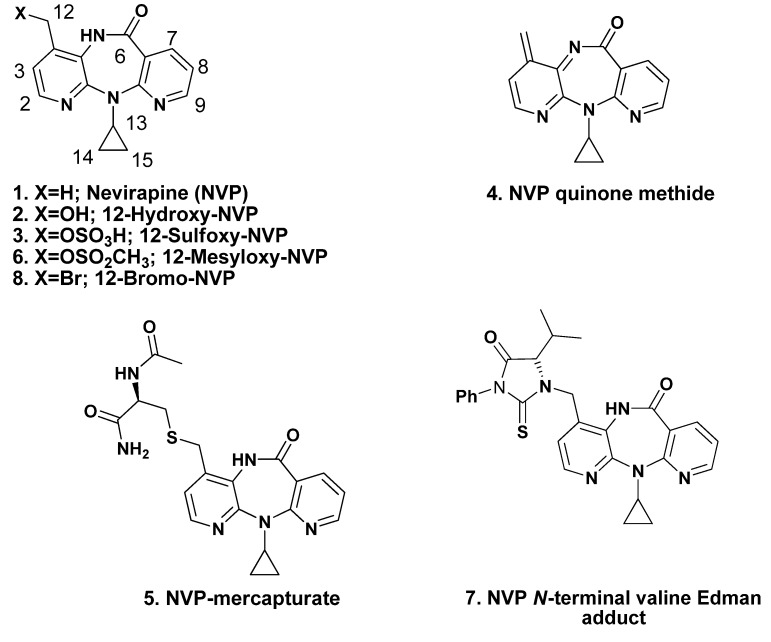
Structures of NVP (**1**), NVP metabolites **2**, **3** and other NVP derivatives **4**–**8** mentioned in the text.

In 1996, nevirapine (NVP, **1**; Figure 1) was the first NNRTI approved by the U.S. FDA. It is presently one of the most prescribed antiretroviral drugs in the developing world, both as a single drug to prevent mother-to-child HIV transmission and as a component of CART [3,4,5,6]. In developed countries, NVP is still a first line choice among initial therapy regimens for children younger than 3 years of age [7]. The high efficacy of the drug, favorable lipid proﬁle [8,9] and suitability for use during pregnancy [10], together with low cost [3], have granted NVP-based regimens a signiﬁcant role in HIV-1 treatment strategies. Nonetheless, the expected increase in worldwide use of NVP, as a result of a more convenient once-daily regimen recently approved by the U.S. FDA [11], should be weighed against toxicity issues. Indeed, besides the above mentioned suggestion of NNRTI carcinogenicity in humans, and although individual susceptibilities to adverse effects differ among patients, NVP administration is associated with a variety of toxic responses, of which the most frequent is skin rash and the most severe is hepatoxicity [12,13,14,15]. The use of NVP also results in the selection of drug resistant mutant strains of HIV-1 [16]. Moreover, while conclusive evidence for NVP carcinogenicity in humans has yet to be presented, long-term administration of the drug to mice and rats resulted in increased incidences of hepatocellular adenomas and carcinomas [17].

While the reasons for the adverse effects of NVP are still unclear, several *in vitro* and *in vivo* data are consistent with the involvement of NVP bioactivation, *via* Phase I oxidation to 12-hydroxy-NVP (**2**, Figure 1) and subsequent Phase II sulfonation to 12-sulfoxy-NVP (**3**, Figure 1), in the onset of toxic events elicited by the parent drug [18,19,20,21]. This Phase II metabolite is a reactive electrophile, and therefore expected to react directly with bionucleophiles (e.g., DNA) yielding covalent adducts. An alternative pathway, involving initial hydrogen sulfate elimination to an NVP quinone methide (**4**, Figure 1), has been proposed to account for the formation of an NVP-mercapturate, through NVP-C12 (**5**, Figure 1), that was identified in the urine of HIV-positive patients administered NVP as part of a standard antiretroviral therapeutic regimen [21]. Recent evidence suggests that the quinone methide may be responsible for NVP-induced liver injury, while 12-sulfoxy-NVP appears to account for NVP-induced skin rash [22,23].

To date, *in vitro* genetic toxicology tests have provided no evidence that NVP is either mutagenic or clastogenic [17]. However, it should be noted that standard mutagenicity assays use exogenous liver-derived metabolic systems. The reactive Phase II conjugates (e.g., sulfates) are thus generated externally, and often have limited capacity to penetrate the target cells due to their increased hydrophilicity compared to the parent compounds. As a result, sulfotransferase-mediated bioactivation is not detected in standard mutagenicity tests [24]. Therefore, a possible explanation for the discrepancy between the negative *in vitro* mutagenicity and the positive *in vivo* rodent carcinogenicity is the involvement of Phase II sulfonation in NVP bioactivation. 

Using 12-mesyloxy-NVP (**6**, Figure 1) as a synthetic surrogate for 12-sulfoxy-NVP (**3**), we demonstrated direct reaction, *in vitro*, with both DNA and the model proteins human hemoglobin and human serum albumin; these experiments allowed us to identify several sites of modification in the bionucleophiles [25,26,27]. Additionally, our data suggested that NVP metabolism to 12-hydroxy-NVP (**2**), followed by Phase II conjugation (e.g., sulfonation) could be a factor in NVP (geno)toxicity. We also confirmed the validity of our synthetic model electrophile **6** to mimic this metabolic activation pathway by using **6** in the preparation of *N*-acetylcysteine (**5**, Figure 1) and glutathione conjugates, through NVP C12 [26], that were identical to those reported by other investigators to be formed *in vivo* or in metabolically competent systems *in vitro* [19,21]. More recently, using *N*-alkyl Edman degradation and by comparison with a synthetic standard also prepared from **6**, we detected and characterized 12-hydroxy-NVP-derived *N*-terminal valine adducts **7** in the hemoglobin of HIV-positive patients on NVP-containing regimens [28].

DNA adduct formation by xenobiotic agents is often mediated by metabolically activated intermediates. Should the DNA lesions remain unrepaired or undergo erroneous repair, this may lead to the initiation of mutagenic and carcinogenic events. The detection of DNA adducts in human tissues is, therefore, an important tool for molecular epidemiology studies, since these adducts act as markers of carcinogen exposure [29]. As such, the availability of synthetic standards and the development of MS-based techniques are of unquestionable relevance to the identification and quantification of these biomarkers at levels expected to be found in humans [30]. Thus, clarifying the possible role of genotoxic pathways at the onset of NVP-induced toxic events requires a good understanding of the patterns of DNA modification by activated NVP metabolites. 

We have previously characterized a number of 2'-deoxyguanosine-, 2'-deoxyadenosine-, and 2'-deoxycytidine-NVP adducts from reaction of **6** with DNA [25]. The potential role of NVP-DNA adduct formation in NVP-induced genotoxicity prompted us to investigate this reaction further, with the aim of establishing whether or not 2'-deoxythymidine (dT) is also a plausible target for 12-hydroxy-NVP-derived electrophilic metabolites. Several lines of evidence indicate that dT adducts may have a significant role in the initiation of mutagenesis and carcinogenesis by certain classes of xenobiotics. For instance, reaction at the exocyclic *O*^4^ of dT (to give *O*^4^-alkyl-dT) has long been associated with the mutagenicity and carcinogenicity of alkylating agents (e.g., *N*-alkyl-*N*-nitroso compounds), due to the poor repair of these lesions [31]. Likewise, bioactivation of the tobacco specific nitrosamine 4-(methylnitrosamino)-1-(3-pyridyl)-1-butanone (NNK) results in the formation of a reactive diazonium ion that alkylates DNA, producing pyridyloxobutyl (POB)-DNA adducts; *O*^2^-POB-dT, the adduct formed upon modification of the exocyclic *O*^2^ of dT, was identified as the most persistent of the POB adducts formed in the lung and liver of male F344 rats treated with NNK and its metabolite, 4-(methylnitrosamino)-1-(3-pyridyl)-1-butanol (NNAL) [32]. Indeed, *O*^4^- and *O*^2^-alkyl-dT adducts appear to be among the most persistent DNA alkylation products in both cultured mammalian cells and animal tissues [33] and may contribute to mutagenesis and carcinogenesis processes long after the original exposure has occurred. In addition, although alkylation of the N3 position of dT typically occurs to a minor extent, both *in vitro* and *in vivo*, N3-alkyl-dT adducts are also considered mutagenic and/or cytotoxic lesions [34,35].

To gain insight into the ability of activated NVP derivatives to form dT adducts, we report herein the synthesis and characterization of NVP-dT adduct standards, and the use of these synthetic standards to identify dT-specific NVP adducts formed in DNA under biologically plausible conditions. 

## 2. Results and Discussion

Given the potential relevance of dT adducts as mutagenic lesions [31,32,33,34,35], and the evidence that the NVP metabolite, 12-hydroxy-NVP (**2**), is bioactivated *in vivo* to electrophilic derivatives that react with bionucleophiles [20,21,23,28], we investigated whether or not NVP-dT adduct formation can occur through the NVP C12. In order to maximize the reliability of adduct characterization, we started with a Buchwald-Hartwig strategy to obtain adduct amounts sufficient for routine spectroscopic analysis. We then proceeded to test our biomimetic electrophile **6**, derived from 12-hydroxy-NVP (**2**), using the previously prepared adduct standards for comparison purposes. 

### 2.1. Synthesis and Structural Characterization of NVP-dT Adduct Standards

**Figure 2:**
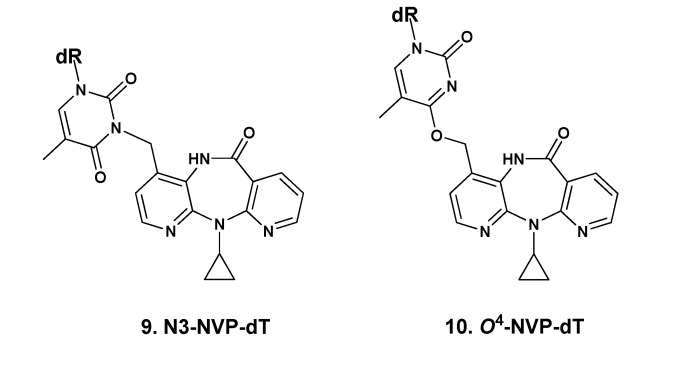
Structures of the NVP adducts, N3-NVP-dT (**9**) and *O*^4^-NVP-dT (**10**), isolated from reaction of 12-bromo-NVP (**8**) with 3',5'-*O*-bis(*tert*-butyldimethylsilyl)-dT under palladium(0) catalysis. dR = 2'-deoxyribosyl.

With the ultimate goal of identifying extra sites of plausible DNA modification upon exposure to activated NVP derivatives, we used an adaptation of the Buchwald-Hartwig strategy of Pd-catalyzed C-N bond formation [25,36] for the preparation of novel NVP-dT adduct standards. The coupling of 3',5'-bis-*O*-(*tert*-butyldimethylsilyl)-dT with 12-bromo-NVP (**8**, Figure 1) was performed for 30 min at 95 °C in toluene, using 2.0 molar equivalents of the protected dT in the presence of tris(dibenzylideneacetone)dipalladium(0) [Pd_2_(dba)_3_, 0.1 equiv] and cesium carbonate (2.0 equiv) for catalyst regeneration. Following desilylation of the 2'-deoxyribosyl (dR) moiety with tetrabutylammonium fluoride, one major adduct, 12-(2'-deoxythymidin-N3-yl)-nevirapine (N3-NVP-dT, **9**, Figure 2), eluting at 18 min under our chromatographic conditions (*cf.* Experimental Section), was isolated in 13% yield by reversed-phase HPLC and fully characterized. 

**Figure 3:**
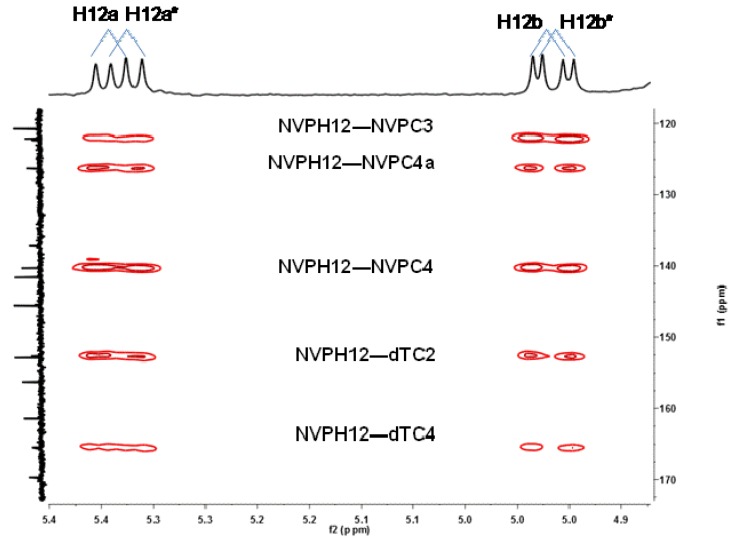
Expanded region of the ^1^H-^13^C HMBC spectrum of N3-NVP-dT (**9**), recorded in methanol-*d_4_*, displaying the connectivities between the geminal NVP-H12 protons and the carbons of the NVP (C3, C4, and C4a) and dT (C2 and C4) moieties. The NVP-H12/dT-C2 and NVP-H12/dT-C4 3-bond connectivities were decisive for structural assignment.

The ^1^H and ^13^C-NMR spectra of **9** confirmed the presence of all the expected resonances from the NVP and dT moieties (*cf.* Experimental Section and Supporting Information, Figures S1–S4). The occurrence of rotamers, which we have also encountered in NVP-amino acid adducts through the NVP C12 [26], was indicated by the duplication of resonances in the ^1^H and ^13^C-NMR spectra (recorded in methanol-*d_4_*) for the NVP H12 protons, all the dT carbons with the exception of C4', and the NVP C3, a position close to the modification site (*cf.* Experimental Section and Supporting Information, Figures S1 and S2). Interestingly, we found no evidence for rotamers when the ^1^H-NMR spectrum was recorded in DMSO-*d_6_*; this suggests that solvent-solute interactions, possibly involving hydrogen bonding with the NVP amido group, play a role in the constrained rotation detected in methanol-*d_4_*. Definite proof of connectivity through dT-N3 was obtained from the HMBC correlations established by the geminal NVP-H12 protons (Figure 3). Thus, in addition to the expected 2- and 3-bond interactions to carbons of the NVP moiety (C3, C4, and C4a), correlations were found with two dT carbons, specifically C2, at 152.7/152.6 ppm, and C4, at 165.5/165.4 ppm, which is unequivocal indication of connectivity through NVP-C12/dT-N3. 

**Scheme 1:**
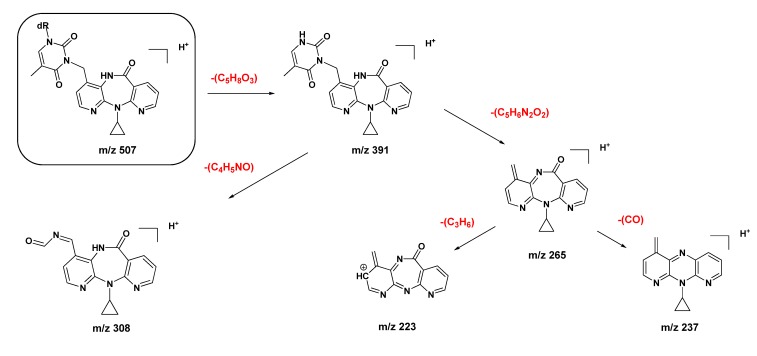
Proposed ESI-MS/MS fragmentation mechanisms for the protonated molecule (*m/z* 507) of N3-NVP-dT (**9**).

The ESI mass spectrum of **9** displayed signals for the protonated molecule at *m/z* 507 and a characteristic fragment ion at *m/z* 391 [(MH_2_-dR)^+^] resulting from loss of the dR moiety (*cf.* Supporting Information, Figure S5). The MS/MS fragmentation pattern was entirely consistent with the assigned structure (Scheme 1; also *cf.* Supporting Information, Figure S6). 

**Figure 4:**
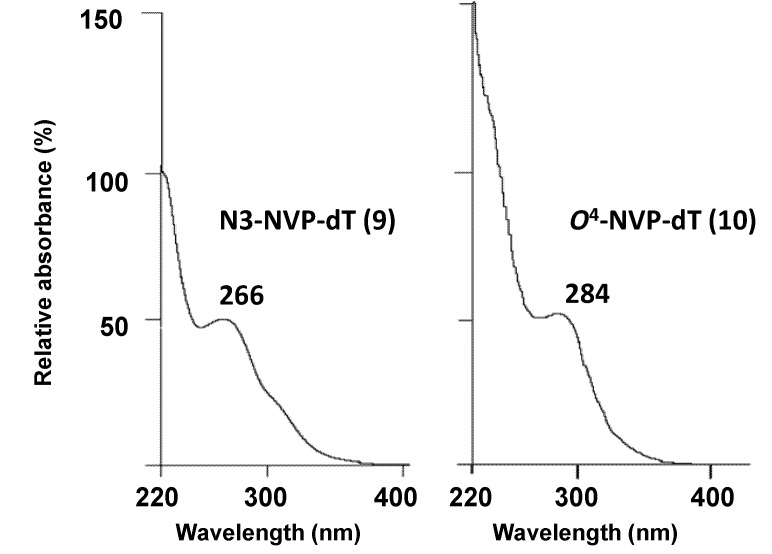
UV spectra of the dT-derived NVP adducts, N3-NVP-dT (**9**) and *O*^4^-NVP-dT (**10**), isolated from reaction of 12-bromo-NVP with 3',5'-bis-*O*-(*tert*-butyldimethylsilyl)-dT under palladium catalysis. The spectra were obtained online, by HPLC with diode array detection, in acetonitrile/0.1% aqueous formic acid.

One additional minor product **10** (Figure 2) of the coupling reaction, eluting *ca*. 3 min earlier than N3-NVP-dT under the conditions used for the HPLC-ESI-MS analysis, was also isolated. Similarly to N3-NVP-dT, the ESI mass spectrum of this species displayed signals for a protonated molecule at *m/z* 507 and a characteristic fragment ion at *m/*z 391 resulting from loss of the dR moiety; this indicated unequivocally that **10** was an NVP-dT adduct. Due to their nucleophilic nature, the two exocyclic oxygens of dT (*O*^2^ and *O*^4^) were potential sites of adduction. We were unable to achieve a definite structural characterization of **10** by NMR analysis because this product was isolated in a very small amount, precluding the establishment of 2- and 3-bond connectivities and also preventing quantification. Nonetheless, and despite the presence of contaminants that masked some signals from the NVP and dT moieties, the ^1^H-NMR spectrum (not shown) yielded significant information. The relevant data are summarized in Table 1, which presents a comparison of UV data and proton chemical shifts for N3-NVP-dT and **10** with the corresponding literature values [37,38,39,40,41] for N3-, *O*^2^- and *O*^4^-methylthymidines. The most noticeable difference between the proton resonances of the two regioisomeric NVP adducts is the downfield shift of the thymidine H6 proton (*ca.* 0.65 ppm) of **10** when compared to **9**. As shown in Table 1, a downfield shift of the H6 proton of dT was observed for *O*^4^-methyl-dT in comparison with the corresponding resonances in N3- and *O*^2^-methyl-dT, which suggests that **10** stemmed from modification at *O*^4^-dT. This regioselectivity in adduct formation is also consistent with the UV profiles exhibited by both adducts. Thus, while adduct **9** had a local maximum at 266 nm, the UV profile of adduct **10** displayed a less defined local maximum at 284 nm (Table 1 and Figure 4). A similar bathochromic shift [41] was observed for *O*^4^-methyl-dT, but not *O*^2^-methyl-dT, when compared to N3-methyl-dT (Table 1). This effect can be explained on the basis of the more extended conjugation established in the thymidine ring upon adduction at the exocyclic *O*^4^ position. 

**Table 1:** Comparison of ^1^H-NMR dT resonances ^[a]^ and UV maxima in adducts **9** and **10** with literature data for methylthymidines.

	N3-methyl-dT ^[b]^	*O*^2^-methyl-dT ^[c]^	*O*^4^-methyl-dT ^[d]^	N3-NVP-dT (9)	*O^4^*-NVP-dT (10)
UV(λ_max_, nm)	267, 235 ^[e]^	259, 237 (pH 1)	280, 241 ^[f]^	266 ^[g]^	284 ^[g]^
		257, 239			
		(pH 13)			
δ (ppm)					
H1'	6.27	6.08	6.24	6.17	6.66
H2'	2.14	2.12–2.17	2.0–2.19	2.50	NA ^[h]^
H3'	3.65–4.76	4.19–4.27	3.84	3.77–3.79	NA
H4'	3.65–4.76	3.70–3.80	4.22	4.26	5.33
H5'	3.55–3.60	3.47–3.66	3.59–3.81	3.50	NA
H6	7.83	7.80	8.01	7.90	8.55
dT-CH_3_	1.87	1.78	1.88	1.90	NA

^[a]^ The spectra were recorded in DMSO-*d*_6_. Chemical shifts are in ppm, downfield from tetramethylsilane; ^[b]^ The NMR data are from Kimura *et al.* [37]; ^[c]^ The NMR and UV data are from Huang *et al.* [38]; ^[d]^ The NMR data are from Miah *et al.* [39]; ^[e]^ The UV data, recorded in water, are from Chang *et al.* [40]; ^[f]^ The UV data, recorded in water, are from Lawley *et al.* [41]; ^[g]^ The spectra were obtained online, by HPLC with diode array detection, in acetonitrile/0.1% aqueous formic acid; ^[h]^ NA, not assigned.

**Scheme 2:**
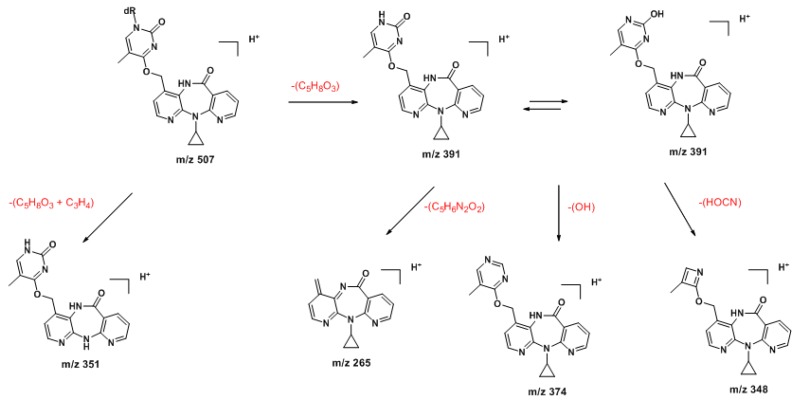
Proposed ESI-MS/MS fragmentation mechanisms for the protonated molecule (*m/z* 507) of *O*^4^-NVP-dT (**10**).

Additional support for the proposed connectivity through *O*^4^-dT in **10** was obtained by tandem MS analysis. Thus, whereas the MS^1^ spectra of the protonated molecules (*m/z* 507) of the two regioisomeric adducts **9** and **10** had only subtle differences, the corresponding MS^3^ spectra clearly displayed distinctive structural features. Indeed, the protonated molecule of the minor adduct was slightly more prone to undergo fragmentation. Besides the characteristic fragment ion resulting from loss of the sugar moiety (*m/z* 391, also observed for **9**), the signal from an additional minor fragment ion at *m/z* 351, stemming from subsequent loss of the cyclopropyl moiety, was observed for adduct **10** (Scheme 2). In addition, and more importantly for characterization purposes, fragmentation of the *m/*z 391 ion from adduct **10** yielded a unique ion at *m/z* 348, stemming from loss of cyanic acid/isocyanic acid with concomitant dT ring contraction (Supporting Information, Figure S8). As indicated in Scheme 2, this fragmentation is consistent with the proposed C12-NVP/*O*^4^-dT connectivity in adduct **10**.

### 2.2. Covalent Modification of dT and DNA with 12-Mesyloxy-NVP (**6**)

We have previously demonstrated direct reaction *in vitro* both with DNA and human blood proteins, using 12-mesyloxy-NVP (**6**, Figure 1) as a synthetic surrogate of the Phase II NVP metabolite, 12-sulfoxy-NVP (**3**) [25,27]. When our initial studies on the ability of activated NVP metabolites to form DNA adducts were conducted, Phase II conjugation of 12-hydroxy-NVP by pathways other than glucuronidation was merely speculative. However, Chen *et al.* [20] subsequently provided evidence for the occurrence of 12-hydroxy-NVP sulfonation *in vivo*, by reporting the LC-MS detection of 12-sulfoxy-NVP in urine and bile samples from female Brown Norway rats administered the parent drug. 

**Figure 5:**
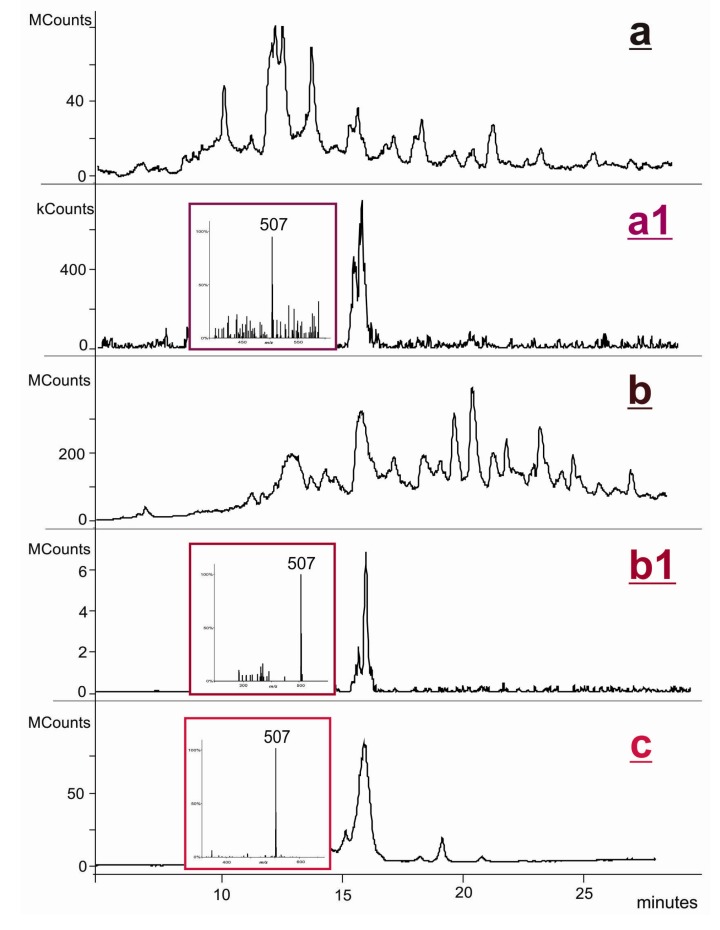
Analysis of the enzymatic hydrolysate of NVP-modified DNA, obtained upon reaction of 12-mesyloxy-NVP (**6**) with salmon testis DNA and subsequent enzymatic hydrolysis to 2'-deoxynucleosides: (**a**) HPLC-ESI-MS total ion chromatogram; (**a1**) extracted ion chromatogram and mass spectrum of the *m/z* 507 ion. Analysis of the reaction mixture obtained upon reaction of 12-mesyloxy-NVP (**6**) with dT: (**b**) HPLC-ESI-MS total ion chromatogram; (**b1**) extracted ion chromatogram and mass spectrum of the *m/z* 507 ion. Analysis of the synthetic standard, N3-NVP-dT (**9**): (**c**) HPLC-ESI-MS total ion chromatogram and mass spectrum of the *m/z* 507 ion.

As indicated above, the validity of our synthetic model electrophile **6** to mimic this metabolic activation pathway has been amply confirmed [19,21,23,25,26,27,28]. Thus, assuming that NVP-induced hepatotoxicity is associated with DNA damage in the liver, the DNA adduct profile formed *in vivo* is expected to follow a pattern similar to that observed *in vitro* upon DNA modification with **6**. To test this biomimetic strategy, we initially conducted a reaction of **6** with dT in DMF/water. Although the yield was very low and no products were isolated, LC-ESI-MS/MS analysis of the reaction mixture revealed the presence of an NVP-dT adduct (MH^+^ at *m/z* 507) that could be identified as N3-NVP-dT (**9**) on the basis of indistinguishable retention times and MS/MS pattern when compared with the corresponding standard under identical elution and ionization conditions (Figure 5). Moreover, these criteria also allowed the identification of the same adduct upon LC-ESI-MS analysis of the enzymatic hydrolysate obtained following the reaction of **6** with salmon testis DNA (Figure 5). These experiments indicate that, although not a major target for DNA adduction by metabolically activated 12-hydroxy-NVP, the N3 of dT can plausibly be modified *in vivo*. Since this position is central to Watson-Crick hydrogen bonding in duplex DNA, covalent modification at N3-dT is expected to interfere with the normal base pairing process, possibly leading to mispairing and/or inhibition of DNA synthesis [42]. In this regard, it is noteworthy that, although produced to a minor extent *in vivo*, N3-alkyl-dT adducts formed by alkylating agents are considered potentially relevant cytotoxic and mutagenic lesions. For instance, N3-ethyl-dT can mispair with dT during *in vitro* DNA replication, resulting in A:T → T:A transversions [35]. Likewise, formation of the N3-Et-dT:dA base pair at the 3'-end of the growing chain has been shown to terminate DNA synthesis [34].

## 3. Experimental

### 3.1. Chemicals

NVP was purchased from Cipla (Mumbai, India). All other commercially available reagents were acquired from Sigma-Aldrich Química, S.A. (Madrid, Spain) and used as received. Whenever necessary, solvents were purified by standard methods [43]. 12-Hydroxy-NVP, 12-mesyloxy-NVP, and 12-bromo-NVP were prepared as described in Antunes *et al.* [25]. 3',5'-*O*-Bis(*tert*-butyldimethylsilyl)-dT was prepared quantitatively by treatment of dT with 10 equivalents of bis(*tert*-butyldimethylsilyl) chloride in pyridine [44].

### 3.2. Instrumentation

#### 3.2.1. Analytical and Semipreparative HPLC

HPLC was conducted on an Ultimate 3000 Dionex system consisting of an LPG-3400A quaternary gradient pump and a diode array spectrometric detector (Dionex, Co., Sunnyvale, CA, USA), equipped with a Rheodyne Model 8125 injector (Rheodyne, Rohnert Park, CA, USA). HPLC analyses were performed with a Luna C18 (2) column (250 mm × 4.6 mm; 5 μm; Phenomenex, Torrance, CA, USA), at a flow rate of 1 mL/min. Semipreparative HPLC separations were conducted with a Luna C18 (2) column (250 mm × 10 mm; 5 μm; Phenomenex) at a flow rate of 3 mL/min. The elution conditions consisted of a 30-min linear gradient of 5%–70% acetonitrile in 0.1% aqueous formic acid, followed by a 2-min linear gradient to 100% acetonitrile, and an 18-min isocratic elution with acetonitrile. The UV absorbance was monitored at 254 nm.

#### 3.2.2. NMR

^1^H-NMR spectra were recorded on a Bruker Avance III 400 spectrometer, operating at 400 MHz. ^13^C-NMR spectra were recorded on the same instrument, operating at 100.62 MHz. Chemical shifts are reported in ppm downfield from tetramethylsilane, and coupling constants (*J*) are reported in Hz; the subscripts *ortho*, *meta*, and *gem* refer to *ortho*, *meta*, and geminal couplings, respectively. Geminal protons are denoted with ‘a’ and ‘b’ labels, and asterisks are used to indicate a second conformer; the proton integrations listed below do not reflect the relative proportions of each conformer. The presence of labile protons was confirmed by chemical exchange with D_2_O. Resonance and structural assignments were based on the analysis of coupling patterns, including the ^13^C-^1^H coupling profiles obtained in bidimensional heteronuclear single quantum coherence (HSQC) and heteronuclear multiple bond correlation (HMBC) experiments, performed with standard pulse programs.

#### 3.2.3. LC-ESI-MS

The liquid chromatography-electrospray ionization-mass spectrometry (LC-ESI-MS) analyses were performed with a ProStar 410 autosampler, two 210-LC chromatography pumps, a ProStar 335 diode array detector, and a 500-MS ion trap mass spectrometer with an ESI ion source (Varian, Inc., Palo Alto, CA, USA). Data acquisition and processing were performed using Varian MS Control 6.9 software. The samples were injected onto the column via a Rheodyne injector with a 20 µL loop. Separations were carried out using a Luna C18 (2) column (150 × 2 mm; 3 µm; Phenomenex) with controlled temperature (30 °C); the mobile phase was delivered at a flow rate of 200 µL/min, using a 5-min isocratic elution of 5% acetonitrile in 0.1% aqueous formic acid, followed by a 30-min linear gradient of 5%–70% acetonitrile, then a 2-min linear gradient to 100% acetonitrile, and a final 8-min isocratic elution with acetonitrile. The mass spectrometer was operated in the positive ESI mode, with the following optimised parameters: ion spray voltage, +5.2 kV; capillary voltage, 20 V; and RF loading, 80%. Nitrogen was used as nebulizing and drying gas, at pressures of 35 and 10 psi, respectively; the drying gas temperature was 350 °C. The tandem mass spectra (MS/MS) were obtained with an isolation window of 2.0 Da, excitation energy values between 0.9 and 1.2 V, and an excitation time of 10 ms.

### 3.3. Syntheses

#### 3.3.1. Palladium-Mediated Coupling of 12-Bromo-NVP (**8**) with 3',5'-*O*-bis(*tert*-butyldimethylsilyl)-2'-deoxythymidine

A suspension of **8** (27 mg, 78 µmol) in toluene (2 mL) was prepared in a screw-capped vial. The palladium catalyst Pd_2_(dba)_3_ (7.5 mg, 8.2 µmol) was added and the mixture was stirred at room temperature for *ca*. 5 min. 3',5'-Bis-*O*-(*tert*-butyldimethylsilyl)-2'-deoxythymidine (75 mg, 159 µmol) and cesium carbonate (52 mg, 160 µmol) were added subsequently and the mixture was stirred at 95 °C for 30 min. Tetrabutylammonium fluoride (1 M solution in THF, 700 µL) was then added to cleave the silyl protection groups, and the mixture was incubated overnight at 37 °C. After centrifugation, the supernatant was decanted and the resulting residue was dissolved in methanol (1.5 mL) and purified by semipreparative HPLC. One adduct was isolated and fully characterized: 

*12-(2'-Deoxythymidin-N3-yl)-nevirapine* (*N3-NVP-dT,* **9**)*.* Obtained in 13% yield (5.1 mg). Retention time, 18 min. UV, λ_max_ 266 nm. ^1^H-NMR (methanol-*d_4_*) δ 8.46 (1H, dd, *J_ortho_* = 4.8, *J_meta_* = 2.0, NVP-H9), 8.10–8.08 (2H, m, NVP-H2 + NVP-H7), 7.89 (1H, s, dT-H6), 7.20 (1H, d, *J_ortho_* = 5.2, NVP-H3), 7.16 (1H, dd, *J_ortho_* = 7.6, *J'_ortho_* = 4.8, NVP-H8), 6.30–6.26 (1H, m, dT-H1'), 5.34 (0.5H, d, *J_gem_* = 14.7, NVP-H12a), 5.33 (0.5H, d, *J_gem_* = 14.8, NVP-H12a*), 4.93 (0.5H, d, *J_gem_* = 14.7, NVP-H12b), 4.92 (0.5H, d, *J_gem_* = 14.8, NVP-H12b*), 4.37–4.35 (1H, m, dT-H3'), 3.89–388 (1H, m, dT-H4'), 3.79–3.67 (3H, m, dT-H5',5" + NVP-H13), 2.28–2.18 (2H, m, dT-H2', H2"), 1.90 (3H, s, dT-CH_3_), 0.91–0.85 (2H, m, NVP-H14 + NVP-H15), 0.44–0.37 (2H, m, NVP-H14 + NVP-H15). ^1^H-NMR (DMSO-*d_6_*) δ 10.17 (1H, bs, NVP-N5H), 8.53–8.52 (1H, m, NVP-H9), 8.11 (1H, d, *J_ortho_* = 5.0, NVP-H2), 8.06 (1H, dd, *J_ortho_* = 7.6, *J_meta_* = 1.7, NVP-H7), 7.90 (1H, s, dT-H6), 7.22 (1H, dd, *J_ortho_* = 7.6, *J'_ortho_* = 4.8, NVP-H8), 6.84 (1H, d, *J_ortho_* = 5.0, NVP-H3), 6.20–6.14 (1H, m, dT-H1'), 5.29 (1H, bs, dT-5'OH/3'OH), 5.22–4.89 (3H, m, NVP-H12 + dT-5'OH/3'OH), 4.26 (1H, bs, dT-H3'), 3.79–3.77 (1H, m, dT-H4'), 3.72–3.50 (3H, m, dT-H5',5" + NVP-H13), *ca.* 2.50 (dT-H2', H2", obscured by the solvent resonance), 1.90 (3H, s, dT-CH_3_), 0.91–0.88 (2H, m, NVP-H14 + NVP-H15), 0.45–0.37 (2H, m, NVP-H14 + NVP-H15). ^13^C-NMR (methanol-*d_4_*) δ 169.7 (NVP-C6), 165.5 (dT-C4), 165.4 (dT-C4*), 161.4 (NVP-C10a), 156.3 (NVP-C11a), 152.9 (NVP-C9), 152.7 (dT-C2), 152.6 (dT-C2*), 145.5 (NVP-C2), 141.6 (NVP-C7), 140.3 (NVP-C4), 137.2 (dT-C6), 137.1 (dT-C6*), 126.3 (NVP-C4a), 122.3 (NVP-C6a), 122.1 (NVP-C3), 122.0 (NVP-C3*), 120.7 (NVP-C8), 111.0 (dT-C5), 110.9 (dT-C5*), 89.0 (dT-C4'), 87.5 (dT-C1'), 87.4 (dT-C1'*), 72.0 (dT-C3'), 71.9 (dT-C3'*), 62.7 (dT-C5'), 62.6 (dT-C5'*), 41.4 (dT-C2'), 41.3 (dT-C2'*), 40.8 (NVP-C12), 30.5 (NVP-C13), 13.1 (dT-CH_3_), 9.7 (NVP-C14/C15), 9.4 (NVP-C14/C15). **MS** *m/z* 507 [MH^+^], 391 [(MH_2_ − dR)^+^].

#### 3.3.2. Reaction of 2'-Deoxythymidine with 12-Mesyloxy-NVP (**6**)

A solution of **6** (1.1 eq., 11 mg, 31 µmol) in THF (1.5 mL) was added to a solution of 2'-deoxythymidine (1.0 eq., 7 mg, 29 µmol) in DMF/H_2_O (2:1; 300 µL). The reaction mixture was incubated at 37 °C for 48 h and subsequently analyzed by LC-ESI-MS.

#### 3.3.3. Reaction of 12-Mesyloxy-NVP (**6**) with DNA

A solution of **6** (5 mg, 14 μmol) in THF (250 µL) was added to a solution of salmon testis DNA (*ca*. 2.5 mg/mL) in 4 mL of 5 mM Bis-Tris and 0.1 mM EDTA (pH 7.1). The mixture was incubated at 37 °C for 60 h. Following removal of the non-bonded materials by extraction with 2 × 1 vol. of ethyl acetate, a second solution of 12-mesyloxy-NVP (5 mg, 14 μmol) in THF (250 µL) was added, and the mixture was re-incubated overnight at 37 °C. The non-bonded materials were removed as indicated above, and the DNA was precipitated by addition of 5 M NaCl (0.1 vol.) and ice-cold ethanol (3 vol.). After centrifugation, the DNA pellet was washed with ice-cold 70% ethanol (2 × 1 vol.) and redissolved in 4 mL of 5 mM Bis-Tris and 0.1 mM EDTA (pH 7.1). The NVP-modified DNA solution was hydrolyzed enzymatically to 2'-deoxynucleosides by treatment with DNAse I, followed by alkaline phosphatase and phosphodiesterase [45]. The adducts were then partitioned into *n*-butanol, that had been presaturated with water, and the *n*-butanol extracts were combined and back-extracted with water, presaturated with *n*-butanol. After the *n*-butanol was evaporated, the residue was redissolved in methanol and analyzed by HPLC-ESI-MS.

## 4. Conclusions

The coupling reaction of 3',5'-bis-*O*-(*tert-*butyldimethylsilyl)-dT with 12-bromo-NVP (**8**) was performed under previously optimized Buchwald-Hartwig conditions [25], allowing the synthesis and structural characterization of two NVP-derived dT adducts, N3-NVP-dT (**9**, major) and *O*^4^-NVP-dT (**10**, minor). These standards were subsequently used to monitor, by LC-ESI-MS/MS, the modification of dT and salmon testis DNA with 12-mesyloxy-NVP (**6**), a synthetic surrogate of the Phase II metabolite 12-sulfoxy-NVP (**3**). N3-NVP-dT, a potentially cytotoxic and mutagenic lesion, was the only dT-specific adduct detected in both instances. The formation of this adduct under our biomimetic conditions suggests that it may also be formed *in vivo*, and play a role in the hepatotoxicity and/or putative hepatocarcinogenicity elicited by NVP. Further work is required to establish if the now available synthetic standard of N3-NVP-dT can be used as a reliable biomarker of NVP-induced (geno)toxicity *in vivo*, thus becoming a molecular tool to help clarify the potential contribution of NVP-containing therapies to the onset of non-AIDS-defining cancers in HIV-positive patients [2].

## Supplementary Materials

*Supporting Information:* NMR and MS (ESI) spectra of adduct **9** and tandem MS (ESI) spectra of adduct **10** are included as Supporting Information. Supplementary materials can be accessed at: http://www.mdpi.com/1420-3049/18/5/4955/s1.
