# Supplementary material for: 2'-Deoxythymidine Adducts from the Anti-HIV Drug Nevirapine
Source: Molecules. 2013 Apr 26;18(5):4955–71. doi: 10.3390/molecules18054955 (PMC6269667; doi:10.3390/molecules18054955)
Supplement: Supplementary file 1 [file molecules-18-04955-s001.pptx]

## Slide 1
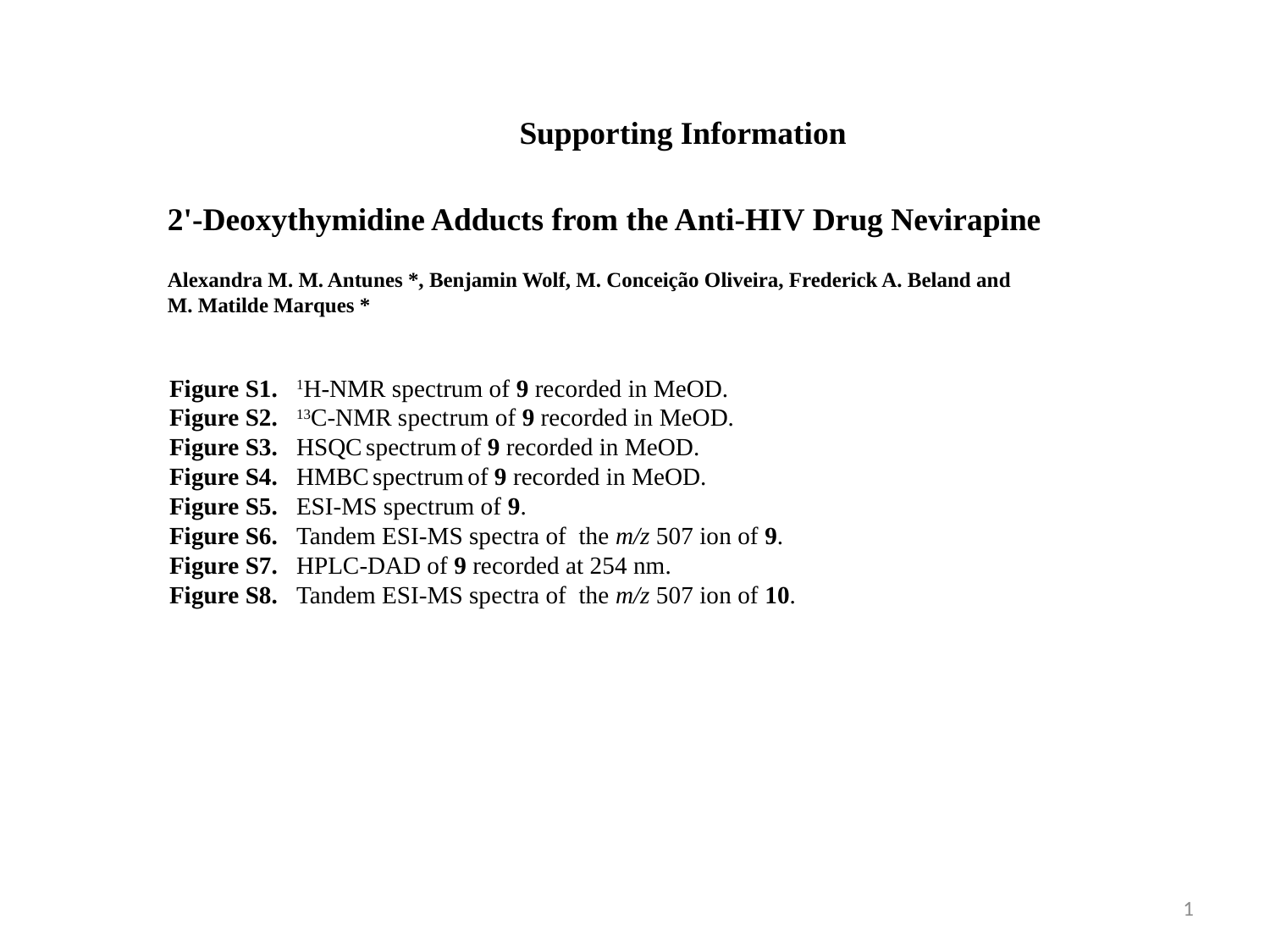

Supporting Information
2'-Deoxythymidine Adducts from the Anti-HIV Drug Nevirapine
Alexandra M. M. Antunes *, Benjamin Wolf, M. Conceição Oliveira, Frederick A. Beland and
M. Matilde Marques *
Figure S1. 	1H-NMR spectrum of 9 recorded in MeOD.
Figure S2. 	13C-NMR spectrum of 9 recorded in MeOD.
Figure S3. 	HSQC spectrum of 9 recorded in MeOD.
Figure S4. 	HMBC spectrum of 9 recorded in MeOD.
Figure S5. 	ESI-MS spectrum of 9.
Figure S6. 	Tandem ESI-MS spectra of the m/z 507 ion of 9.
Figure S7. 	HPLC-DAD of 9 recorded at 254 nm.
Figure S8.	Tandem ESI-MS spectra of the m/z 507 ion of 10.
1

## Slide 2
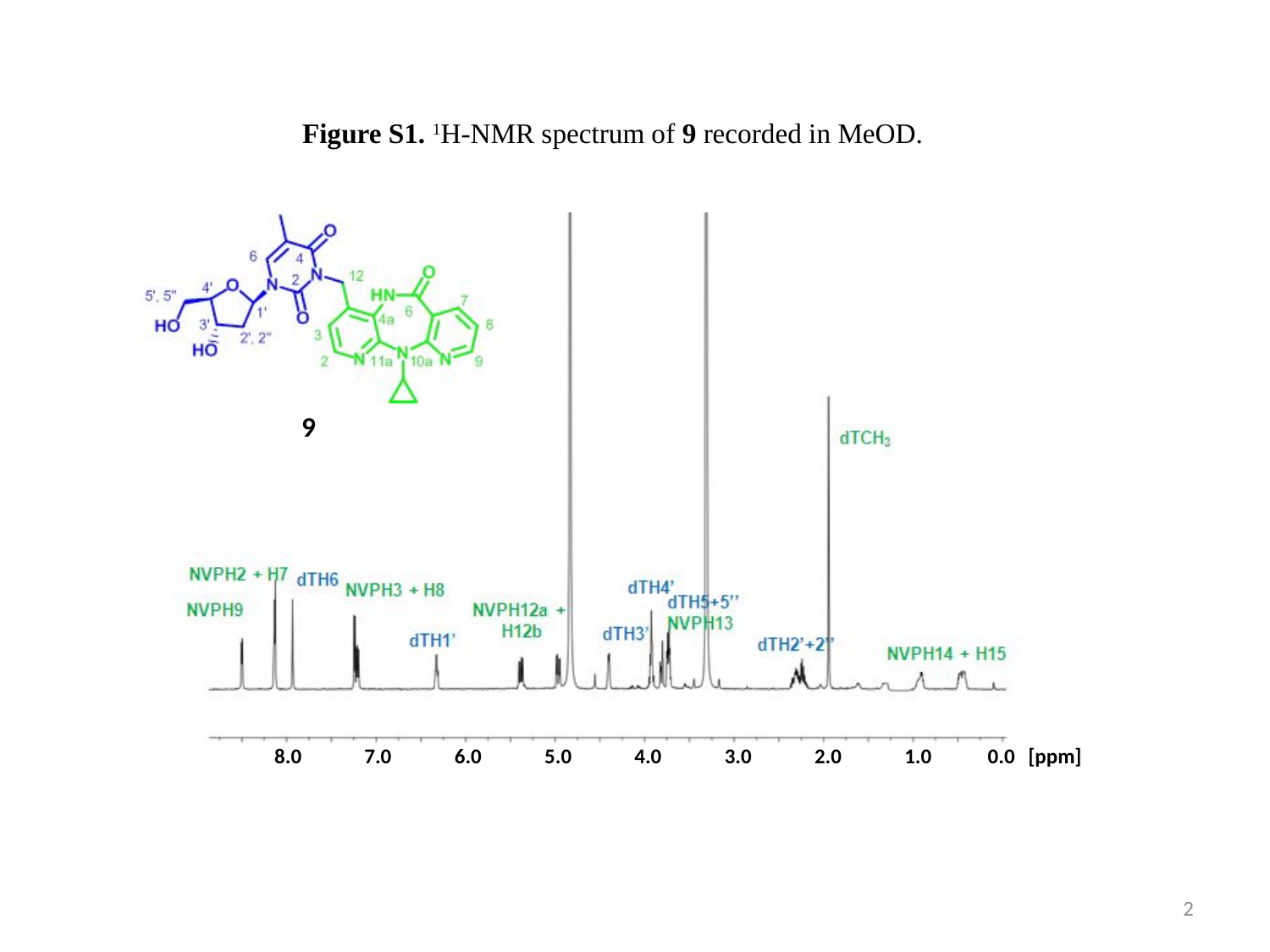

Figure S1. 1H-NMR spectrum of 9 recorded in MeOD.
9
8.0
7.0
6.0
5.0
4.0
3.0
2.0
1.0
0.0
[ppm]
2

## Slide 3
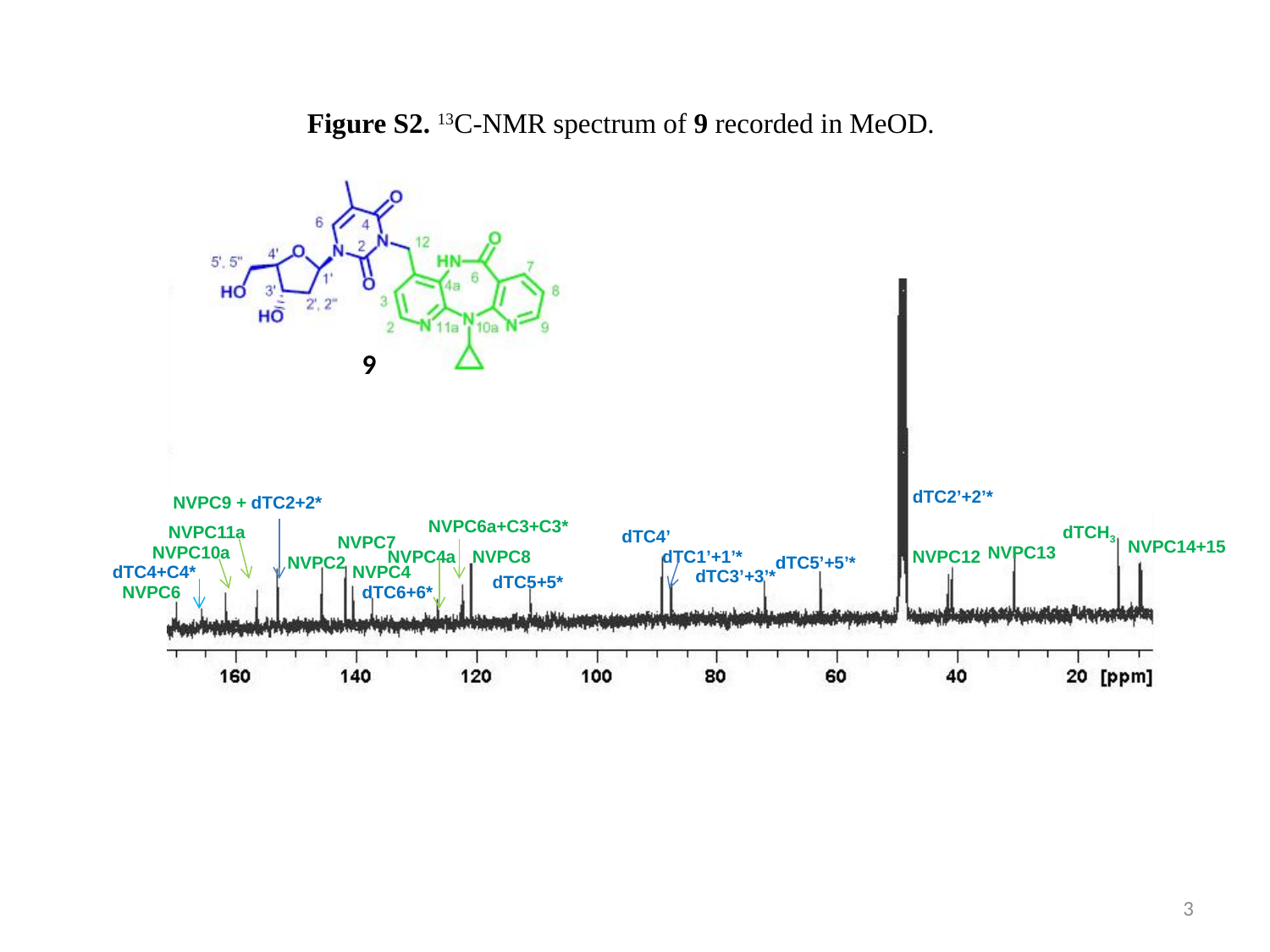

Figure S2. 13C-NMR spectrum of 9 recorded in MeOD.
9
dTC2’+2’*
NVPC9 + dTC2+2*
NVPC6a+C3+C3*
NVPC11a
dTCH3
dTC4’
NVPC7
NVPC14+15
NVPC10a
NVPC13
NVPC4a
NVPC8
dTC1’+1’*
NVPC12
NVPC2
dTC5’+5’*
dTC4+C4*
NVPC4
dTC3’+3’*
dTC5+5*
NVPC6
dTC6+6*
3

## Slide 4
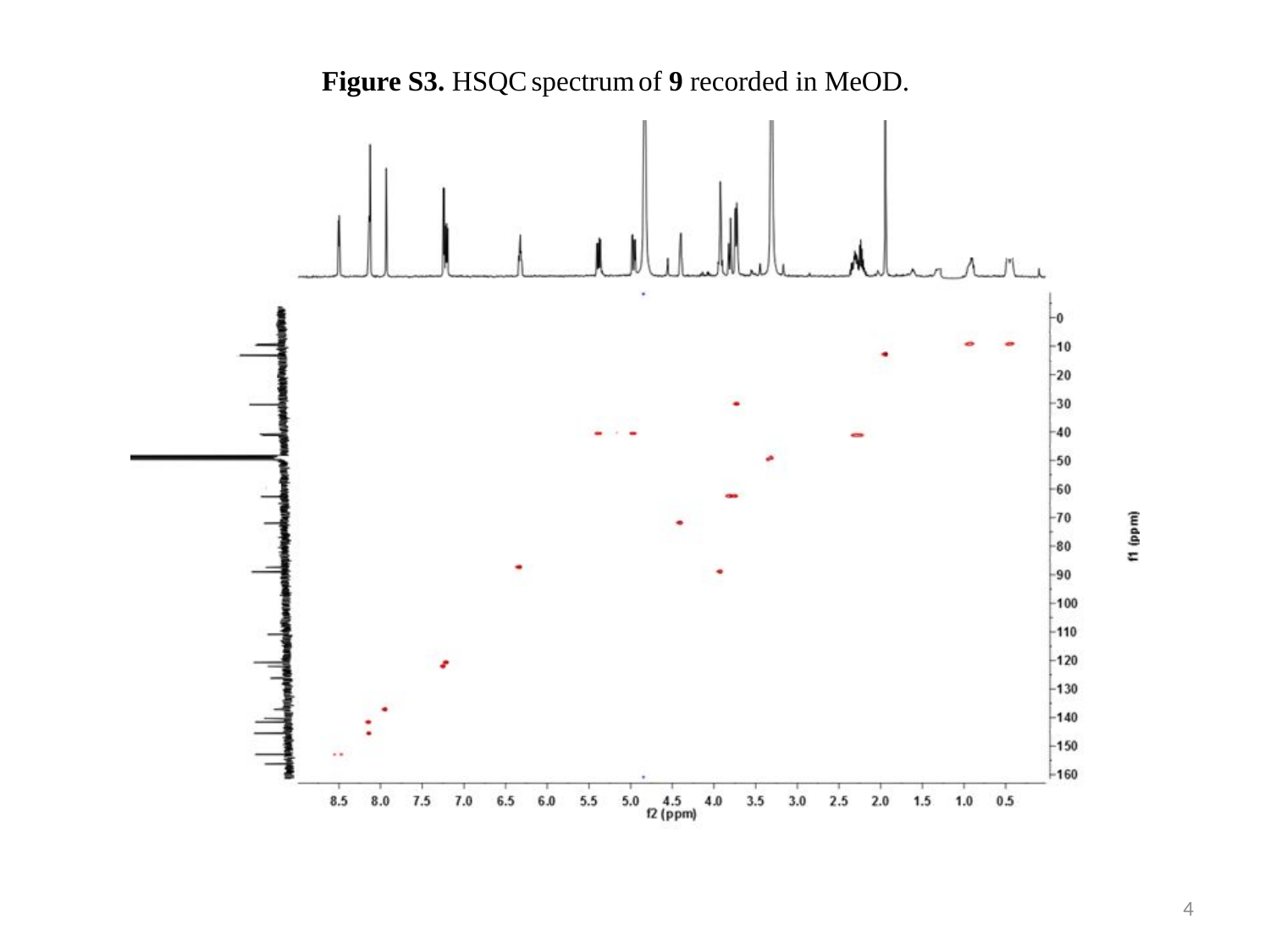

Figure S3. HSQC spectrum of 9 recorded in MeOD.
4

## Slide 5
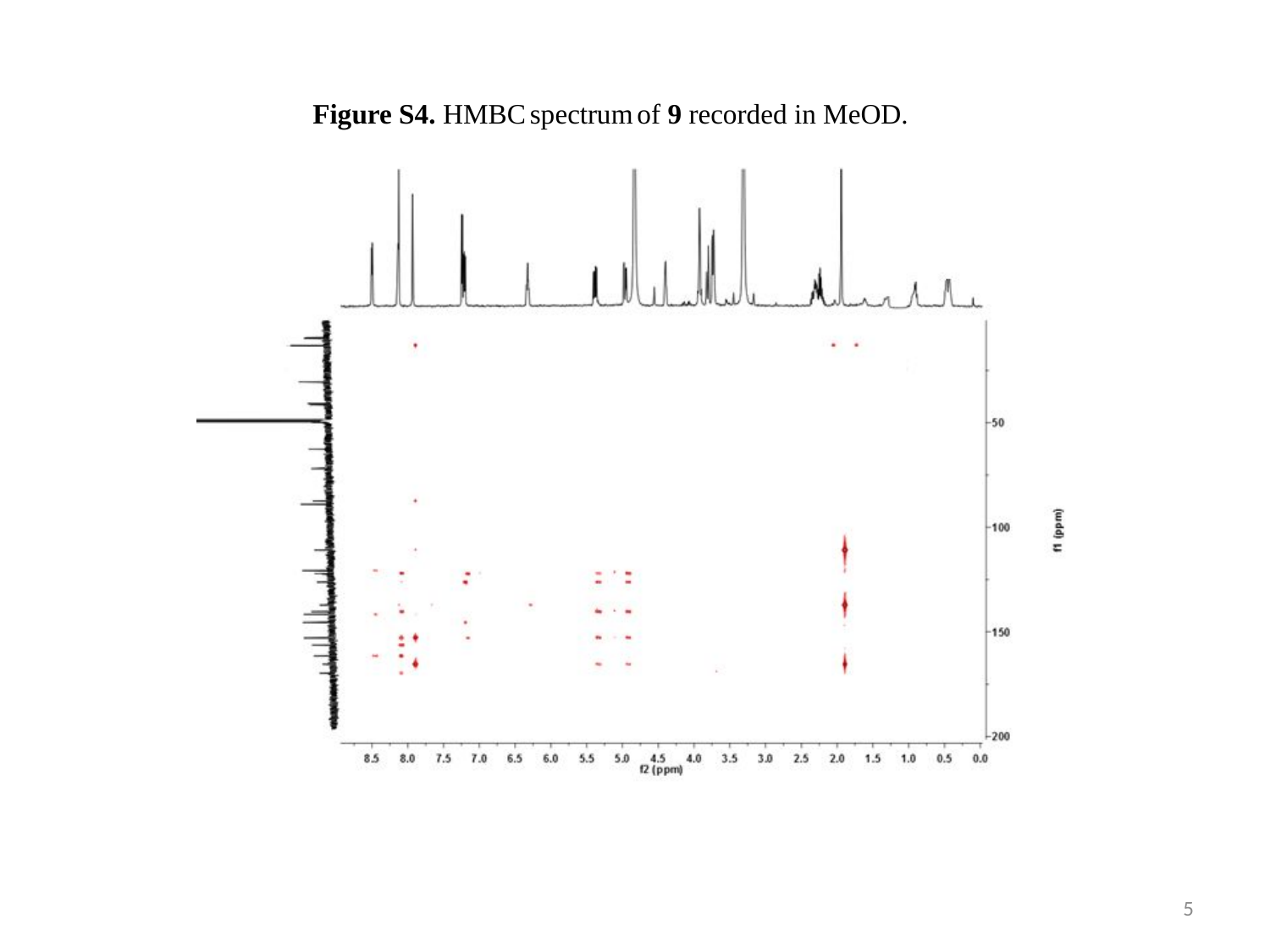

Figure S4. HMBC spectrum of 9 recorded in MeOD.
5

## Slide 6
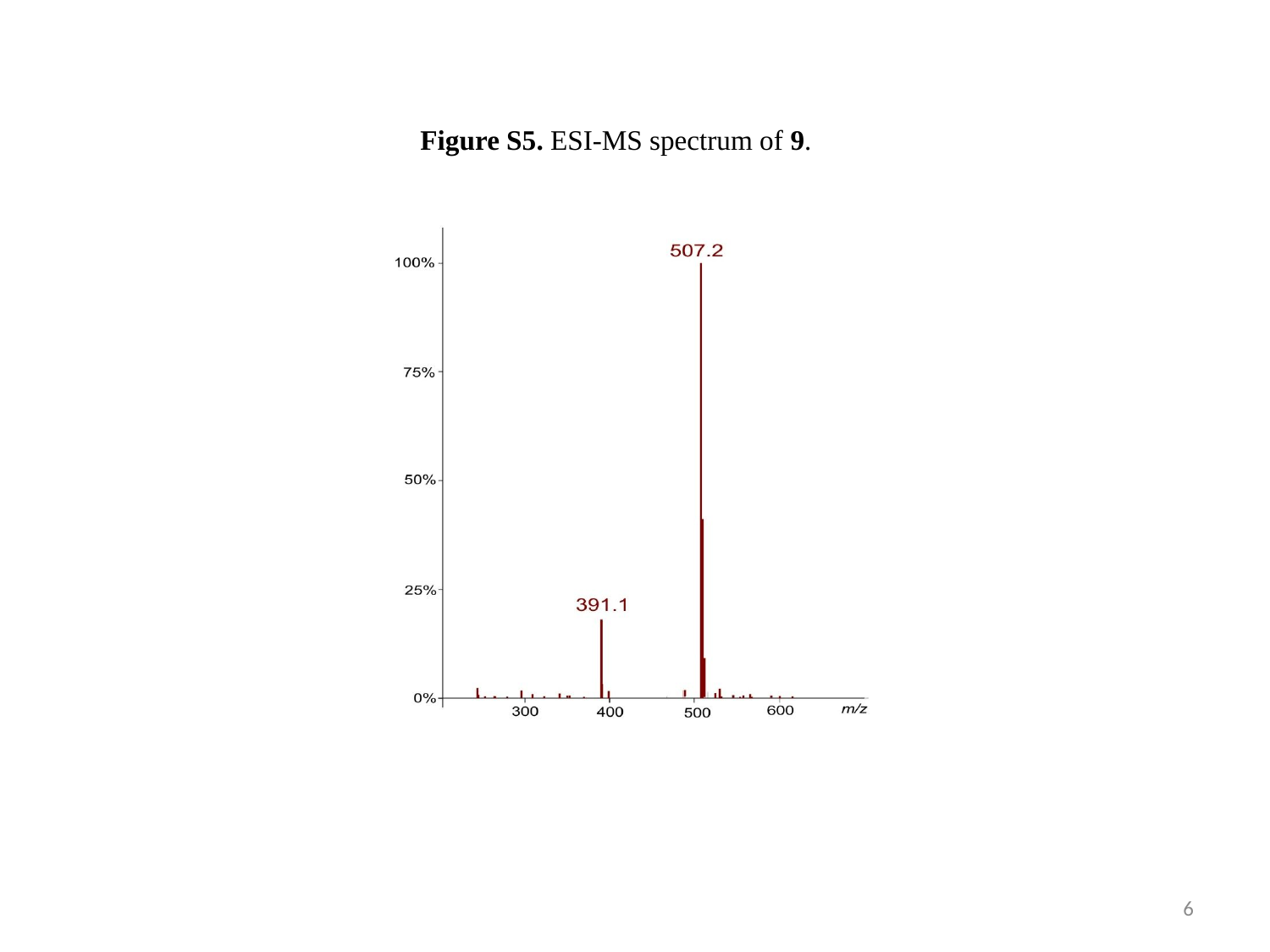

Figure S5. ESI-MS spectrum of 9.
6

## Slide 7
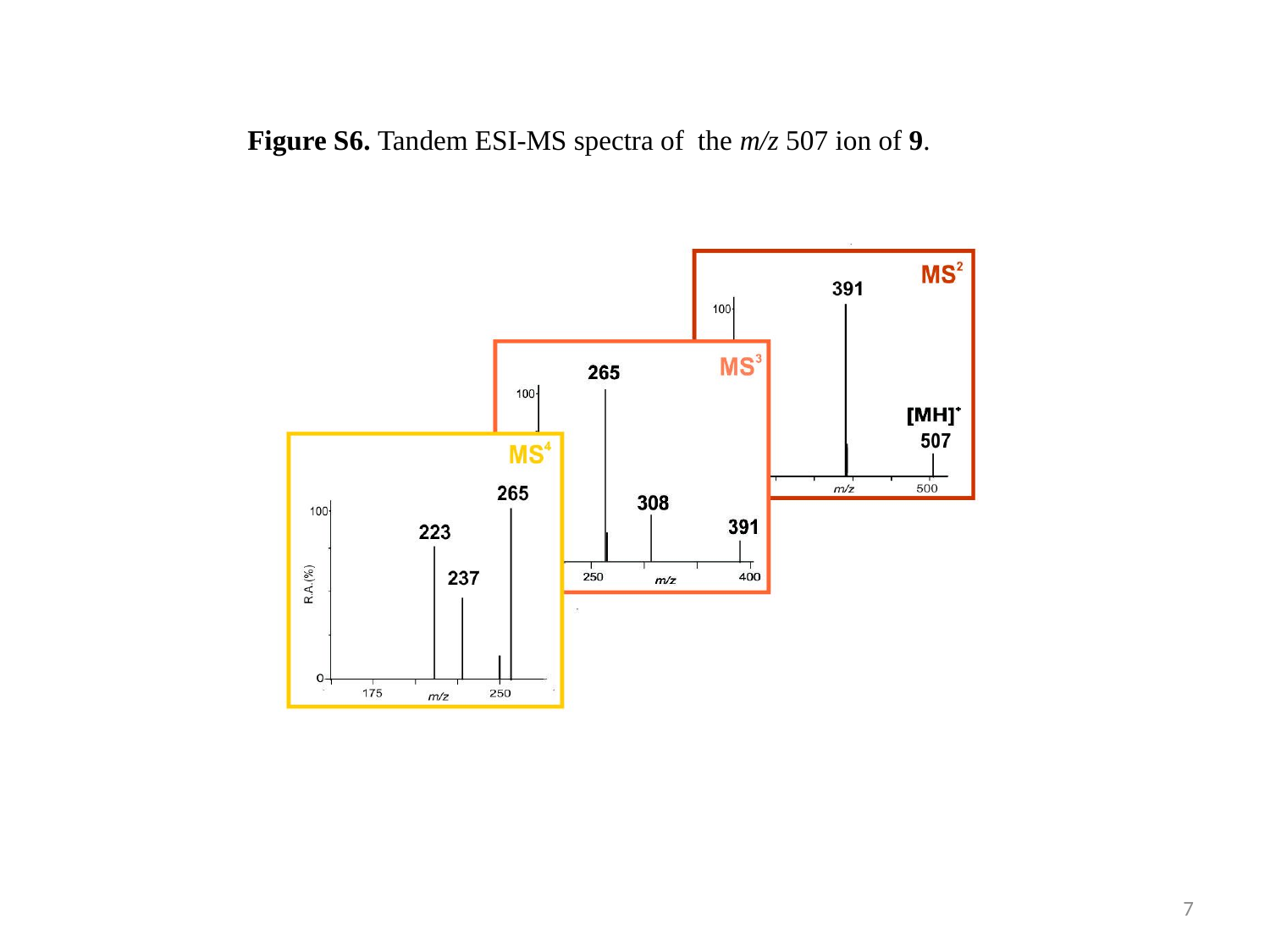

Figure S6. Tandem ESI-MS spectra of the m/z 507 ion of 9.
7

## Slide 8
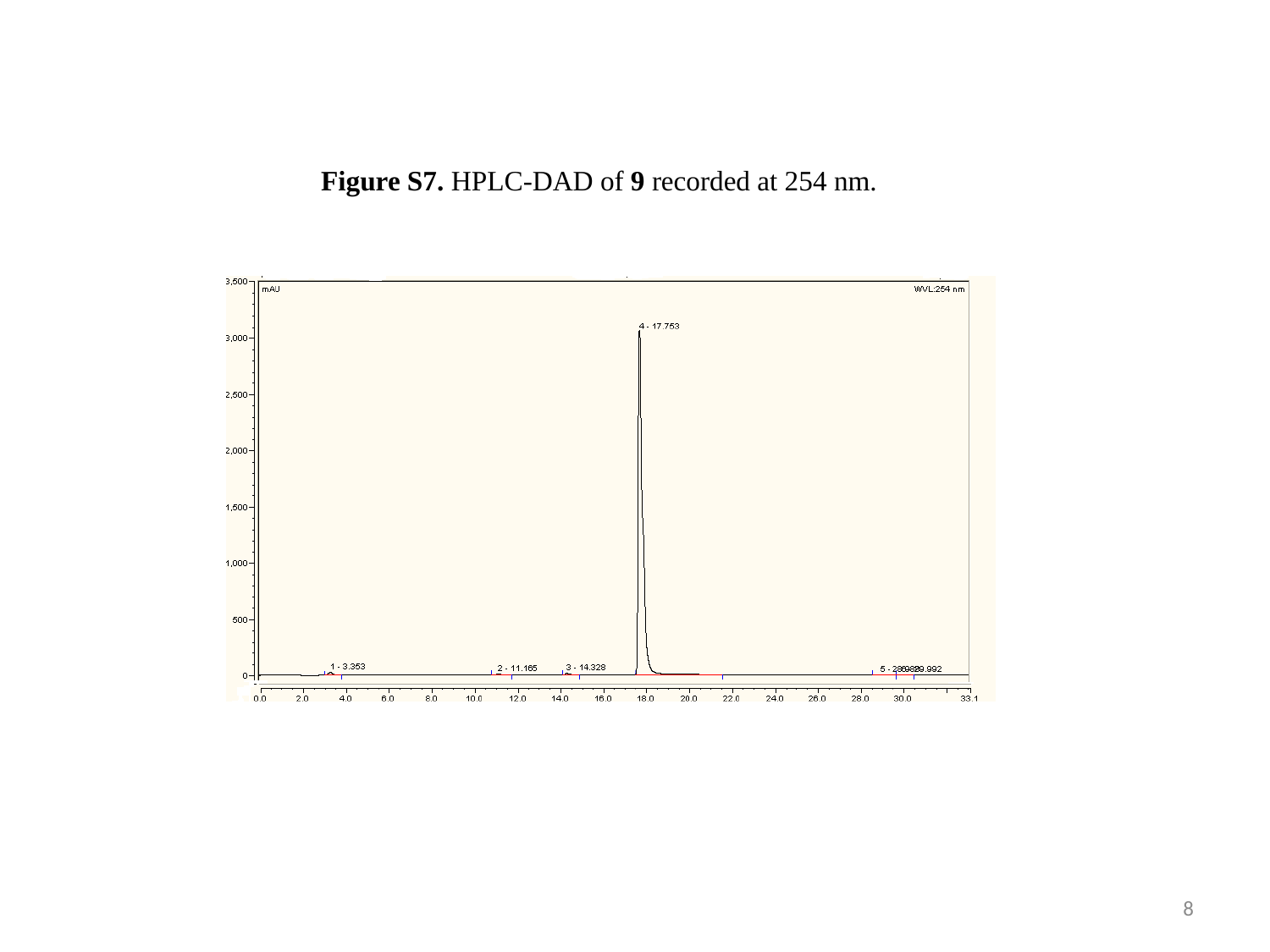

Figure S7. HPLC-DAD of 9 recorded at 254 nm.
8

## Slide 9
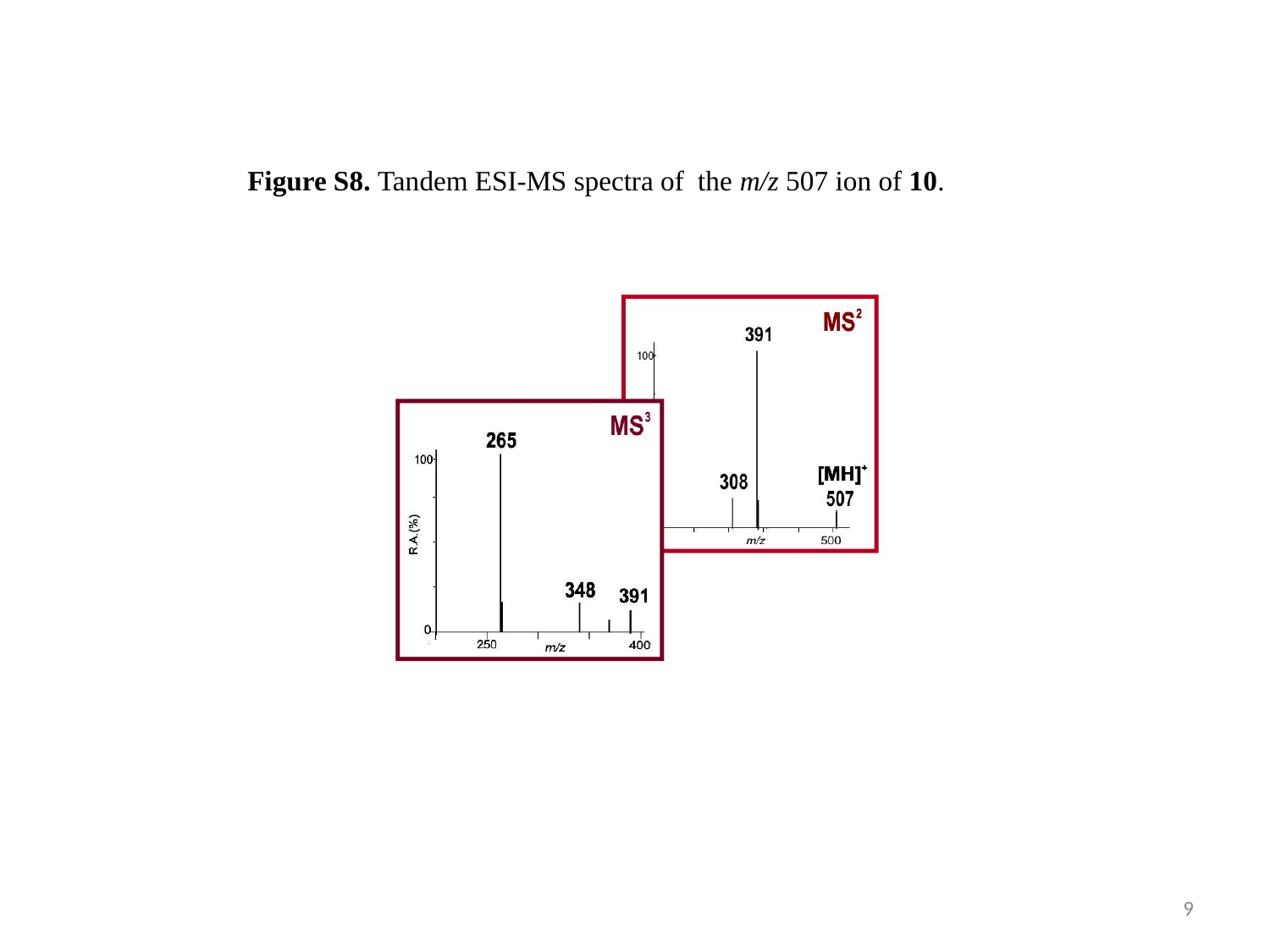

Figure S8. Tandem ESI-MS spectra of the m/z 507 ion of 10.
9
